# Frailty detection in older adults via fractal analysis of acceleration signals from wrist-worn sensors

**DOI:** 10.1007/s13755-023-00229-8

**Published:** 2023-06-27

**Authors:** Antonio Cobo, Ángel Rodríguez-Laso, Elena Villalba-Mora, Rodrigo Pérez-Rodríguez, Leocadio Rodríguez-Mañas

**Affiliations:** 1 Centre for Biomedical Technology, Universidad Politécnica de Madrid, Autopista M-40 km. 38, 28223 Pozuelo de Alarcón, Madrid Spain; 2 CIBER de Bioingeniería, Biomateriales y Nanomedicina, Instituto de Salud Carlos III, Avda. Monforte de Lemos, 28029 Madrid, Madrid Spain; 3 CIBER de Fragilidad y Envejecimiento Saludable, Instituto de Salud Carlos III, Avda. Monforte de Lemos, 28029 Madrid, Madrid Spain; 4 Fundación para la Investigación Biomédica, Hospital Universitario de Getafe, Ctra. Madrid-Toledo km. 12.5, 28905 Getafe, Madrid Spain; 5 Department of Signal Theory, Communications, Telematics, and Computing, Universidad Rey Juan Carlos, Camino del Molino, 5, 28942 Fuenlabrada, Madrid Spain; 6 Geriatrics Department, Hospital Universitario de Getafe, Ctra. Madrid-Toledo km. 12.5, 28905 Getafe, Madrid Spain

**Keywords:** Frailty syndrome, Unobtrusive monitoring, Fractal analysis, Time series, Accelerometry, Smartwatch

## Abstract

****Purpose**:**

Frailty is a reversible multidimensional syndrome that puts older people at a high risk of adverse health outcomes. It has been proposed to emerge from the dysregulation of the complex system dynamics of physiologic control systems. We propose the analysis of the fractal complexity of hand movements as a new method to detect frailty in older adults.

****Methods**:**

FRAIL scale and Fried’s phenotype scores were calculated for 1209 subjects—72.4 (5.2) y.o. 569 women—and 1279 subjects—72.6 (5.3) y.o. 604 women—in the pubicly available NHANES 2011–2014 data set, respectively. The fractal complexity of their hand movements was assessed with a detrended fluctuation analysis (DFA) of their accelerometry records and a logistic regression model for frailty detection was fit.

****Results**:**

Goodness-of-fit to a power law was excellent (R$^{2}>0.98$). The association between complexity loss and frailty level was significant, Kruskal–Wallis test (df = 2, Chisq = 27.545, p-value $<0.001$). The AUC of the logistic classifier was moderate (AUC with complexity = 0.69 vs. AUC without complexity = 0.67).

****Conclusion**:**

Frailty can be characterized in this data set with the Fried phenotype. Non-dominant hand movements in free-living conditions are fractal processes regardless of age or frailty level and its complexity can be quantified with the exponent of a power law. Higher levels of complexity loss are associated with higher levels of frailty. This association is not strong enough to justify the use of complexity loss after adjusting for sex, age, and multimorbidity.

## Introduction

Data from the World Bank for 2019 show that 92.29% of the women and 84.68% of the men in the European Union (EU) survived to age 65 [1, 2]. According to current predictions, they will live for approximately 20 more years (21.8 years, in the case of women and 18.3 years, in the case of men) [3]. The World Health Organization defines healthy aging as “the process of developing and maintaining the functional ability that enables well-being in older age” ([4], p. 28). In other words, healthy aging involves remaining free of functional limitations and disability until very close to the end of life. However, according to current predictions, people 65 years old will enjoy only half of their 20 years ahead without functional limitations and disability. This scenario concerns 91,496,893 people 65 years old and older in the EU [5], representing 20.46% of the overall European population [6]. Before developing any severe functional limitations and becoming disabled, older people may transition from robustness to a state of increased vulnerability known as frailty that can last for several years before eventually transitioning into disability [7]. Frailty is a multidimensional syndrome that makes homeostasis difficult even under exposure to low power stressors [8]. Consequently, frail people are at a high risk of adverse outcomes, including twice the risk of disability than non-frail older adults [9], as well as falls, hospitalization, permanent institutionalization, and death [8, 10–12]. Besides its detrimental effect on people’s functional ability and health, frailty also impacts health care systems economies. In 2012, frail people in the top 10% group of Medicare individual spending beneficiaries were just 4% of the Medicare population in the USA. Despite they represented such a small proportion of the population, they accounted for 43.9% of the total potentially preventable spending [13]. Frailty is a well established concept in geriatrics, however, there is still some debate on how to conceptualize it [14]. One of the most validated and used approaches is the frailty phenotype, initially described by Fried et al. [7], which considers that frailty is manifested through five pre-defined signs and symptoms: exhaustion; wasting; and reduction of activity, strength and speed. Because some of these components need to be measured, one with a dynamometer, attempts have been made to use questions to ascertain even those designed to be measured; one of these questionnaires is the FRAIL scale [15]. Fortunately, frailty, in contrast to a disability, can be reversed [16–18]. Clinical interventions based on physical exercise proved to be effective in restoring frail people to robustness [16, 17, 19]. These exercise-based interventions are particularly effective if delivered at the early stages of the functional decline process and if the older adult remains engaged in the care program [20]. Thus, identifying frail people at early stages is paramount. Some authors suggest that frailty is an emergent property with no specific cause, arising from the complex system dynamics of physiologic control systems [21]. This hypothesis is consistent with the fact that dysregulation signs in so many different physiologic systems have shown associations with frailty. Ghachem et al. [21] even observed the amount of dysregulation to be more important than the particular identity of the systems involved. As a result, people’s ability to adapt to daily stressors degrades and functional decline arises [22]. Rector et al. [23] highlight two different paradigms that model the adaptive behaviors of physiological systems, namely, critical slowing down (CSD) and loss of complexity (LoC). Either of these paradigms makes a different assumption. CSD-based indicators model resilient, adaptive responses as low variability fluctuations around an equilibrium point, whereas LoC-based indicators model resilient adaptability as complex fluctuations following a power law across time scales like fractals do [22, 24]. The appropriate paradigm may depend on the homeostatic role of the target variable—i.e., regulated vs. effector variables—with effector variables behaving consistently with the LoC paradigm [23]. The complexity of the dynamic fractal behavior of many effector variables has in fact been observed to degrade with aging and disease [22]. For instance, LoC under normal aging conditions has been observed in heart rate (HR) [25], blood pressure [26], respiratory cycle [27], stride interval [28], and postural sway dynamics [29, 30]. Besides, less complex HR showed increased cardiovascular mortality in heart failure and ischemic heart disease patients [31], and older fallers showed less complex stride–stride intervals [32]. Moreover, greater complexity values have been observed to be associated to greater ability to perform adaptive tasks [22]. The overall effect of the progression of frailty and the subsequent functional decline is the degradation of people’s ability to keep on with their activities of daily living (ADLs). Hence, we wonder whether dysregulation in behavioral variables, such as physical activity (PA), can be used as a proxy for the overall dysregulation in physiologic control systems. Previous works on the complexity of PA suggest this variable behaves consistently with the LoC paradigm (i.e., complexity decreases in the presence of frailty). In several cross-sectional studies, older people with more complex PA patterns have shown lower levels of frailty [23], disability [23], and fear of falling [33]. In contrast, older people with high variance in PA have not shown high levels of frailty and disability, as would have been expected under the CSD paradigm [23]. In the same line, older people with less complex PA patterns at baseline have shown a higher risk of becoming frail and disabled [34] or even dying [34, 35] in follow-up studies. There is even evidence of a significant correlation between longitudinal changes in the complexity scores of PA and changes in balance and mobility performance after 4 weeks of home-based physical exercise [36]. In particular, Rector et al. [23] quantified PA complexity as the multiscale entropy (MSE) of the acceleration time series recorded by an Inertial Measurement Unit (IMU) on people’s chests. IMUs are a kind of sensors comprising an accelerometer and a gyroscope, which measure linear acceleration and angular speed, respectively. Then, the fluctuations of PA over time can be represented as a time series of activity counts [37]. Samples in a time series of activity counts are usually defined as the sum of raw acceleration values over epochs of a particular duration after removing frequency components outside the human movement spectrum [37]. IMUs based on Micro-Electromechanical Systems (MEMS) are tiny enough to come embedded in usual commercial devices such as smartphones, smartwatches, and fitness trackers. Lately, wrist-worn devices such as smartwatches and fitness trackers have consolidated their place as consumer goods and are a much more natural and socially accepted option than wearing sensors on the chest or on a belt around the waist. However, people move their arms and hands even during the course of sedentary activities. Thus, it is unclear whether time series of acceleration or activity counts from wrist-worn devices are a proper representation of people’s PA. Despite that, Li et al. [34] found a relationship between the complexity of people’s non-dominant hand movements and the future onset of frailty. They observed that people with lower complexity scores at baseline had a higher risk of becoming frail over the next years. However, the potential use of hand-movement complexity to assess and diagnose the present level of frailty in older adults has not been explored yet. In the present paper we study whether the LoC in the fluctuations of the activity counts from wrist-worn sensors is capable of detecting frailty. We obtained the self-similarity exponent ($\alpha$) of the hand-movement signals by means of a detrended fluctuation analysis (DFA) which is a common method for non-stationary time series [35, 38]. A value of $\alpha = 1$ corresponds to pink noise, which has been observed in many natural phenomena showing fractal behaviors [39]. In fact, values of $\alpha$ close to 1.0 have been observed to reflect healthy fractal complexity in HR signals [24]. As the value of $\alpha$ decreases, the long range correlations in the corresponding stochastic processes tend to disappear until their samples become completely uncorrelated (white noise) for $\alpha = 0.5$. On the other hand, as the value of $\alpha$ increases, the corresponding stochastic processes lose roughness and become Brownian noise for $\alpha = 1.5$; which means that departures from pink noise in either direction are associated to complexity loss. Thus, we used the departures from pink noise in $\alpha$ as a measurement of complexity loss in hand-movement fluctuations.

## Methods

We conducted a retrospective observational cross-sectional study on data from the National Health and Nutrition Examination Survey (NHANES). This survey is a program of studies designed to assess the health and nutritional status of adults and children in the United States [40]. It examines a nationally representative sample of about 5000 civilian, non-institutionalized persons each year [40]. Most of the data set is in the public domain and is available on the Internet. This is a retrospective study of anonymized data in the public domain, and no ethical approval was required.

### Participants

Subjects in the 2011–2014 NHANES waves were included in the present study if they:

- met ALL the following INCLUSION CRITERIA:
  - subjects 20 years old or older,
  - subjects with available data from the Physical Activity Monitor (PAM) in the summary data files, and
  - subjects wearing the PAM on their non-dominant hand,
- did not meet ANY of the following EXCLUSION CRITERIA:
  - subjects with less than 2 days of valid PAM data,
  - subjects without enough data to compute their frailty score,
  - subjects with a history of stroke, and
  - subjects taking antidepressants, antiparkinsonian drugs, or acetylcholinesterase inhibitors.

### Apparatus

NHANES 2011–2014 relied on wrist-worn PAMs to collect PA information (CDC, 2020). In particular, they used the ActiGraph model GT3X+, manufactured by ActiGraph of Pensacola, FL [40]. NHANES participants over 3 y.o. were asked to wear the PAM for seven consecutive days to collect objective information on 24-h movement when awake and asleep [40]. In particular we used data from the Physical Activity Monitor—Header (PAMHD) and Physical Activity Monitor—Day (PAXDAY) data files to test the inclusion criteria; and we used the triaxial acceleration measurements summarized at the minute level in the Physical Activity Monitor—Minute (PAXMIN) data file to compute PA complexity. NHANES 2011–2014 reports them in Monitor-Independent Movement Summary (MIMS) units, “which is a non-proprietary, open-source, device-independent universal summary metric developed by researchers at Northeastern University (John et al. 2019)”.

On the other hand, NHANES 2011–2014 relied on examination tests and responses to different questionnaires to collect functional information [40]. Some of them were conducted during enrollment in a controlled environment in a mobile examination center (MEC) while some others were conducted during an interview in the subjects’ homes. In particular, we used data from the Demographics Data (DEMO), Body Measures Data (BMX), Muscle strength-Grip test Data (MGX), Mental Health—Depression screener Questionnaire (DPQ), the Physical Functioning Questionnaire (PFQ), Physical Activity Questionnaire (PAQ), the Blood Pressure & Cholesterol Questionnaire (BPQ), the Diabetes Questionnaire (DIQ), the Medical Conditions Questionnaire (MCQ), the Kidney Conditions—Urology Questionnaire (KIQ_U), and the Weight History Questionnaire (WHQ), Prescription Medication Questionnaire (RXQ_RX) and Muscle Strength Data (MSX).

### Procedure

We used DFA to compute the self-similarity index ($\alpha$) of the participants’ non-dominant hand movements. We used the triaxial acceleration measurements summarized at the minute level to build a time series for each participant. In accordance with the methodology described by Raichlen et al., we considered as valid those days containing between 10 and 20 h of valid wake time [35]. Samples labeled as sleep, non-wear, or unknown as well as samples with reliability flags and missing MIMS values were removed from the data set. The remaining valid wake minutes across all valid days were combined in a single time series for each participant. As stated by Raichlen et al. [35], “Ma et al. (29) have shown that this method does not significantly alter the final results of the DFA analysis in time-series data sets.” DFA was performed on each subject using the nonlinearTseries package in R version 4.1.2 [41] “with 25 window sizes ranging from 10 minutes to 7 hours in length”. Then, we computed the Manhattan distance between $\alpha$ and 1 (i.e., $d = |1 - \alpha |$) as a measurement of complexity loss.

**Fig. 1:**
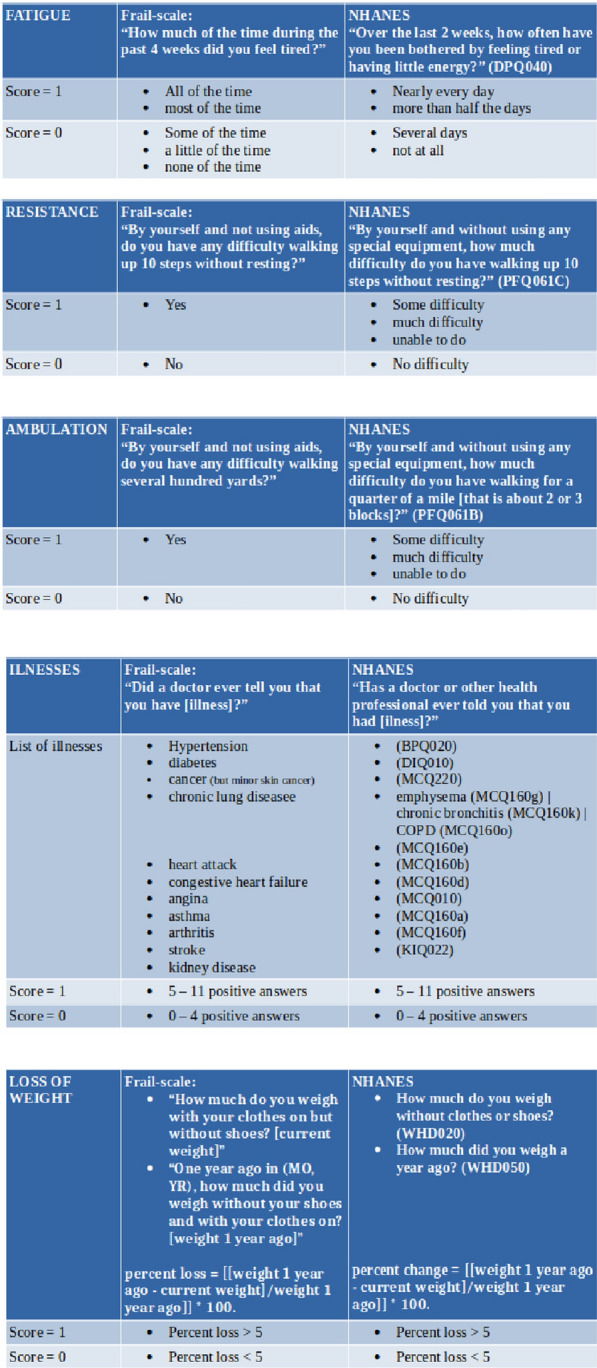
Mapping of NHANES 2011–2014 variables on to the FRAIL scale

We appraised the participants’ frailty status by assessing their frailty phenotype and scoring their FRAIL scale. Since data for neither of those scales were explicitly collected in NHANES 2011–2014, we mapped NHANES variables on to them. The mapping on to the items of the FRAIL scale was quite straightforward as described in Fig. 1. Individuals were classified as robust, if they did not score any item; prefrail, if they scored 1 or 2 items; and frail, if they scored 3 or more items.

In relation to the frailty phenotype, the loss of weight and the exhaustion items were calculated in the same fashion than for the FRAIL scale (with the addition that the loss of weight had to be unintentional). The grip strength item was calculated as the lowest quintile of the maximum strength measured with a handgrip dynamometer at three attempts in both hands (items MGXH1T1, MGXH1T2, MGXH1T3, MGXH2T1, MGXH2T2, MGXH2T3), for age (below and above 65 years old), sex and BMI ($\le 24;>24, \le 26;>26, \le 28; >28$) strata.

The presence of the PA item was defined as being in the lowest quintile of kilocalories expended per week according to a questionnaire based on the Global Physical Activity Questionnaire (GPAQ) of the WHO, which includes questions on number of days per week and minutes each day of daily activities, leisure time activities, and sedentary activities (items PAQ610, PAD615, PAQ625, PAD630, PAQ640, PAD645, PAQ655, PAD660, PAQ670, PAD675). Quintiles were calculated by strata of age (below and above 65 years old) and sex. Actually, the worst quintile in all strata was 0 expenditure.

Gait speed, the last item of the frailty phenotype, was not measured in the NHANES 2011–2014, which nevertheless included questions on difficulty walking for a quarter of a mile (PFQ060B), walking up 10 steps without resting (PFQ060C) and walking from one room to another on the same level (PFQ060H; possible answers for all of them: no difficulty, some difficulty, much difficulty, unable to do). We used NHANES 2001–2002, which included these same questions and measured usual-pace gait speed at 20 ft. for individuals 50 years or older (the use of a walker or cane was permitted for the timed walk), to assess which of them detected better the lowest quintile of gait speed, stratified by age (below and above 65 years old), sex and height (below and above 173 and 159 cm for men and women, respectively). The combination of the answers having some or much difficulty and being unable to walk for a quarter of a mile, offered the best aggregate of accuracy (0.79 $-95\%$ CI 0.77; 0.81), sensitivity (0.51 $-95\%$ CI 0.45; 0.56) and specificity (0.85 $-95\%$ CI 0.83; 0.87). Taking away the category ‘some difficulty’ increased accuracy at the expense of diminishing sensitivity notably.

In the same fashion than for the FRAIL scale, individuals who did not score any items were considered robust; those who scored 1 or 2, prefrail; and those who scored 3 or more, frail.

Multimorbidity was measured as a count of ten conditions referred by the patient as having been diagnosed by a doctor: diabetes; coronary heart disease, including angina and heart attacks/myocardial infarction; congestive heart failure; ictus; asthma; chronic obstructive pulmonary disease, including emphysema and current chronic bronchitis; rheumatoid arthritis; current liver conditions; weak/failing kidneys; and cancer, up to four of them and including lymphomas and leukemias. The multimorbidity count was calculated even when up to three answers to individual conditions were missing.

### Analysis

NHANES uses a complex, multistage probability design to sample the civilian, noninstitutionalized population residing nationwide in the U.S. (in the 50 states and D.C.) [40]. NHANES sampling weights and complex design were taken into account by using the survey package in R version 4.1.2. [41].

#### Fractal-like behavior

We tested the fractal-like behavior of the participants’ hand movements by assessing the goodness-of-fit of their fluctuations across time scales to a power law. We estimated the linearity (R$^{2}$) of the relationship between the logarithmic of the fluctuations and the values of the expected fitted line. Then we used the svymean function to estimate the mean value of R$^{2}$ and the confint function to estimate its 95% confidence interval. Finally, we used a scatterplot to look for any relationships between goodness-of-fit and the value of self-similarity.

#### Associations between frailty and complexity loss

We tested the associations between frailty status and complexity loss with non-parametric Kruskal–Wallis tests instead of one-way ANOVA tests because the assumption of an unbounded outcome did not hold. We also used non-parametric Kruskal–Wallis tests with the Bonferroni correction for pairwise comparisons between frailty levels. We used the svyranktest function in the survey package to compute the Kruskal–Wallis tests and the p.adjust method to compute the Bonferroni correction.

#### Building the logistic regression models

We built two logistic regression models for people over 65 years old, with a binary outcome (robust = 0 vs. pre-frail/frail = 1) based on the frailty phenotype in one model and the FRAIL scale in the other one. We used age, sex, multimorbidity and complexity loss as candidate covariates.

We assessed the linearity of continuous covariates (complexity loss, age, and multimorbidity) and identified potential nonlinear transformations by visual inspection of their corresponding smoothed scatter plots [42] with the svysmooth function in the survey package. We fitted a bivariate model for each covariate, applying the corresponding nonlinear transformation when appropriate, and assessed the significance of their coefficients. We then fitted a model with sex and age as initial covariates and subsequently added multimorbidity and complexity loss one at a time. We assessed the significance of their coefficients and built our main effects models (FRAIL model and FRIED model) by retaining the significant ones.

We fitted the models with the svyglm function in the R survey package with a quasibinomial family. We tested the overall significance of all the coefficients in the models with multivariable Wald tests and multivariable modified Wald tests. We conducted the multivariable Wald tests with the wald.test function in the R aod package. We conducted the multivariable modified Wald tests by manually computing the F statistic as described by Hosmer et al. [43] and by using the pf function to compute the p-value.

We checked for interactions between complexity loss and each of the other covariates after centering the variables.

The results of the model building process are summarized in “Appendix”.

#### Goodness of fit and predictive performance

We assessed goodness-of-fit with Archer and Lemeshow’s extension of the decile of risk test for complex sample surveys [44]. We adapted the Si and Pritchard’s working version of R code [45] to our use case according to Damico’s suggestions [46]. We assessed the power of the test by evaluating the sample size and the frequency of the outcome according to Hosmer’s et al. rule of thumb: $n \ge 500\, and\, 0.25 \le n1/n \le 0.75$ [47].

We assessed the predictive performance of the models by computing the area under their receiver operating characteristic curves (AUC) with the roc.curve function in the PRROC package. We interpreted the results according to the following rule of thumb [47]:

- $AUC = 0.5$ suggests no discrimination,
- $0.5< AUC < 0.7$ is considered poor discrimination,
- $0.7 \le AUC < 0.8$ is considered acceptable discrimination,
- $0.8 \le AUC < 0.9$ is considered excellent discrimination,
- $AUC \ge 0.9$ is considered outstanding discrimination.

## Results

### Sample

A total of 19,931 people participated in NHANES 2011–2014—mean age (standard deviation) 31.4 (24.5) y.o., 10,072 women. Among them, 6722 participants—47.7 (17.2) y.o., 3276 women—were included in the present study. The 13,209 excluded subjects were 23.2 (23.5) y.o., 6796 women. Data to compute the FRAIL scale scores were available for 6,480 of them—47.0 (17.1) y.o., 3159 women; on the other hand, data to assess the frailty phenotype were available for 3906 of them—56.8 (15.4) y.o., 1952 women. Both the FRAIL scale and the frailty phenotype were computed for 3664 subjects. Table 1 describes the overall, Frail scale, and Fried phenotype samples.

**Table 1:** Descriptive statistics for the overall sample of NHANES participants included in the present study (left) as well as for the FRAIL (center) and Fried phenotype (right) subsets

	Overall sample		FRAIL scale sample		Fried phenotype sample	
	Total	$\ge 65$ y.o.	Total	$\ge 65$ y.o.	Total	$\ge 65$ y.o.
Sample size	6722	1326	6480	1209	3906	1279
Age	47.7 (17.2)	72.6 (5.3)	47.0 (17.1)	72.4 (5.2)	56.8 (15.4)	72.6 (5.3)
Women	3276	626	3159	569	1952	604
Robust	n/a	n/a	4307 (66.5 %)	697 (57.7%)	1389 (35.6%)	555 (43.4 %)
Prefrail	n/a	n/a	2043 (31.5%)	463 (38.3%)	2326 (59.5%)	641 (50.1%)
Frail	n/a	n/a	130 (2.0%)	49 (4.1%)	191 (4.9%)	83 (6.5%)

For each of the three samples, figures are provided for the total amount of subjects and for the subset of older adults 65 years old and older

Table 2 shows the confusion matrix for the FRAIL scale and the frailty phenotype for people in the sample 50 years old and older.

**Table 2:** Confusion matrix between the outcomes of applying the FRAIL scale and the Fried phenotype, as defined in the methods section, to the subset of people 50 years old and older in our overall sample

Fried phenotype	FRAIL scale		
	Robust	Prefrail	Frail
Robust	1171	210	0
Prefrail	564	641	44
Frail	0	66	53

### Fractal-like behavior

**Fig. 2:**
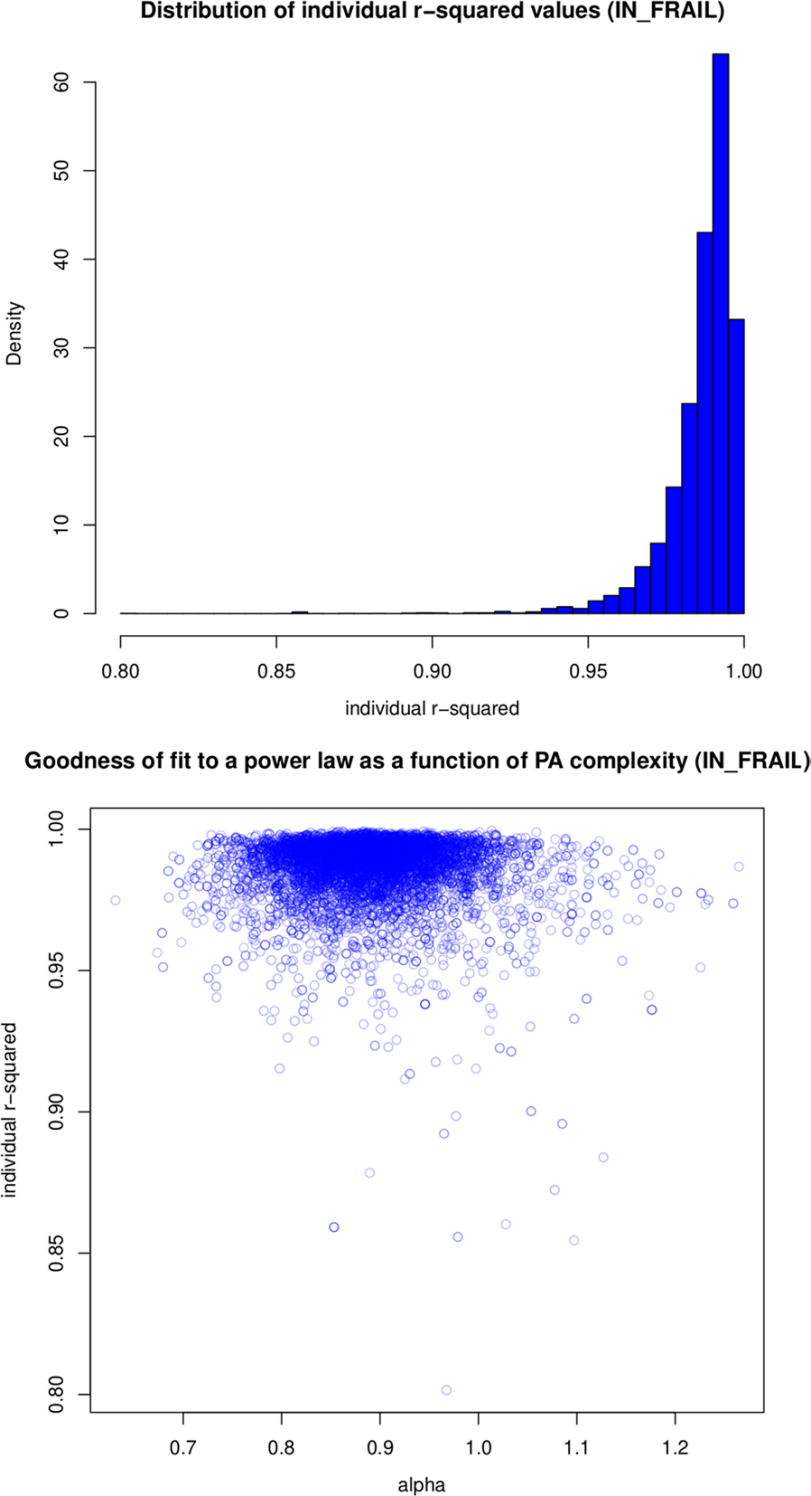
On the top, distribution of individual R$^{2}$ values (FRAIL scale). On the bottom, goodness-of-fit as a function of self-similarity scores (FRAIL scale). No relationship is observed between the two variables

The population represented by the participants with the FRAIL scale assessed showed R$^{2}$ = 0.9865 (0.0002) with a 95% CI = (0.9861, 0.9868) and a distribution as shown in Fig. 2 (top). The scatterplot in Fig. 2 (bottom) does not show any relationships between goodness-of-fit and self-similarity scores.

**Fig. 3:**
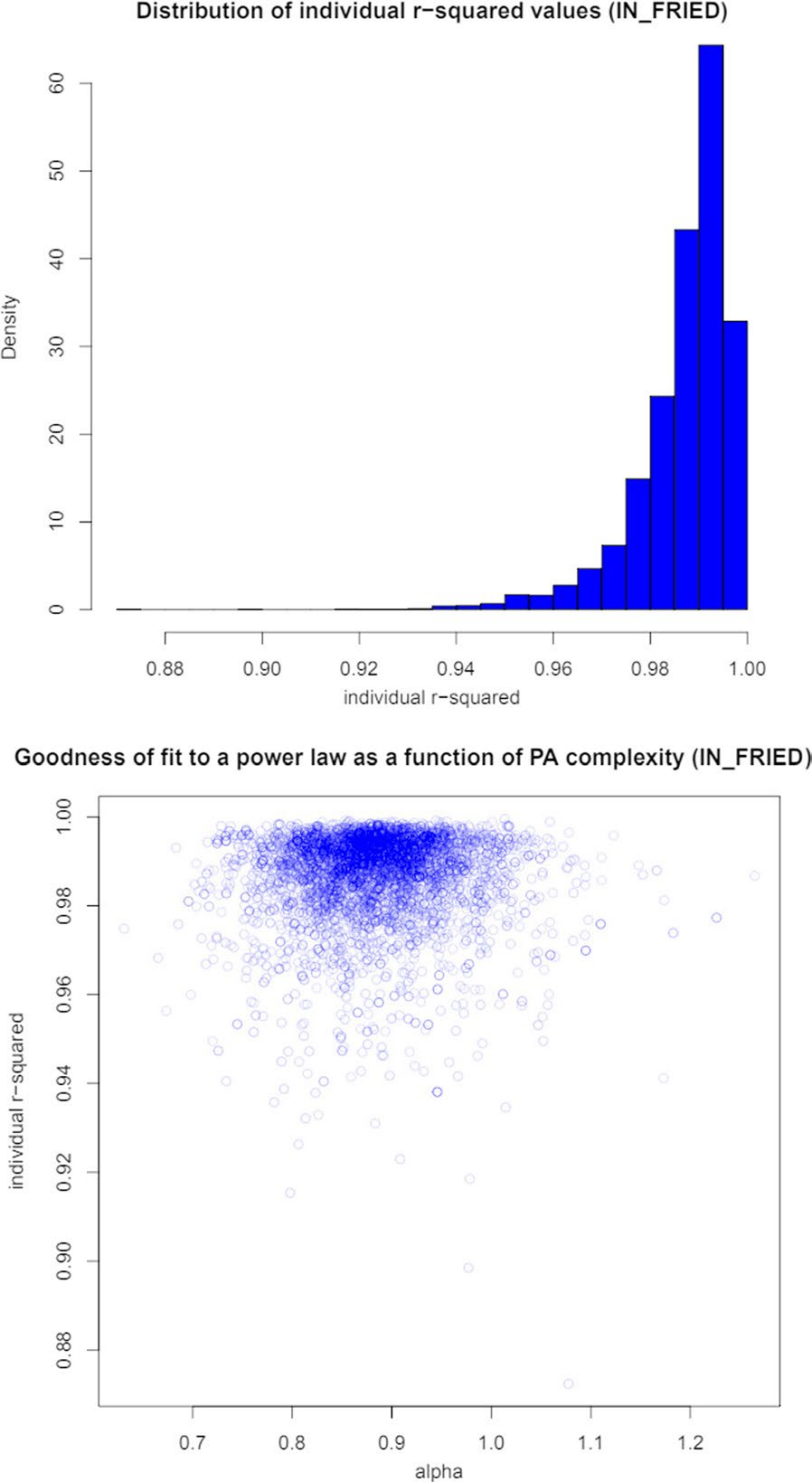
On the top, distribution of individual R$^{2}$ values (Fried phenotype). On the bottom, goodness-of-fit as a function of self-similarity scores (Fried phenotype). No relationship is observed between the two variables

The population represented by the participants with the frailty phenotype assessed showed R$^{2}$= 0.9870 (0.0002) with a 95% CI = (0.9865, 0.9874) and a distribution as shown in Fig. 3 (top). The scatterplot in Fig. 3 (bottom) does not show any relationship between goodness-of-fit and self-similarity scores.

Table 3 shows the results for the assessment of the fractal behavior of hand movements across age groups for the overall sample and the FRAIL scale and Fried phenotype subsets. Goodness-of-fit is excellent in all of them (R$^{2}> 0.98$)

**Table 3:** Results for the assessment of the fractal behavior of hand movements across age groups, namely, young adults (left), middle-aged adults (center) and older adults (right)

	20–49 y.o.	50–64 y.o.	65+ y.o.
Overall	R$^{2}$ = 0.9844 (0.0004)	R$^{2}$ = 0.9864 (0.0004)	R$^{2}$ = 0.9853 (0.0004)
	(0.9837, 0.9851)	(0.9856, 0.9872)	(0.9844, 0.9861)
FRAIL scale	R$^{2}$ = 0.9860 (0.0002)	R$^{2}$ = 0.9871 (0.0004)	R$^{2}$ = 0.9872 (0.0004)
	(0.9855, 0.9865)	(0.9863, 0.9879)	(0.9864, 0.9881)
Fried phenotype	R$^{2}$ = 0.9864 (0.0004)	R$^{2}$ = 0.9872 (0.0004)	R$^{2}$ = 0.9871 (0.0004)
	(0.9855, 0.9873)	(0.9864, 0.9880)	(0.9863, 0.9879)

Results are reported for the overall sample (top) the FRAIL scale subset (center) and the Fried phenotype subset (bottom). Average values, standard deviations, and confidence intervals for the linearity of the log–log fluctuations of hand movements across different time scales are reported for each subset

Table 4 shows the results for the assessment of the fractal behavior of hand movements across levels of functional status for the FRAIL scale and the Fried phenotype subsets. Goodness-of-fit is excellent in all of them (R$^{2}> 0.98$).

**Table 4:** Results for the assessment of the fractal behavior of hand movements across different frailty levels, namely, robust (left), prefrail (center), and frail (right)

	Robust	Prefrail	Frail
FRAIL scale	R$^{2}$ = 0.9866 (0.0002)	R$^{2}$ = 0.9863 (0.0003)	R$^{2}$ = 0.9871 (0.0012)
	(0.9861, 0.9870)	(0.9857, 0.9868)	(0.9846, 0.9896)
Fried phenotype	R$^{2}$ = 0.9874 (0.0003)	R$^{2}$ = 0.9868 (0.0004)	R$^{2}$ = 0.9850 (0.002)
	(0.9867, 0.9881)	(0.9864, 0.9873)	(0.9810, 0.9890)

Results are reported for the FRAIL scale (top) and the Fried phenotype (bottom) subsets. Average values, standard deviations, and confidence intervals for the linearity of the log–log fluctuations of hand movements across different time scales are reported for each subset

### Associations between frailty and complexity loss

Associations between frailty status and complexity loss for the overall population, regardless of age, were statistically significant for both the FRAIL scale (df = 2, Chisq = 56.966, p-value $< 0.001$) and the frailty phenotype (df = 2, Chisq = 68.343, p-value $< 0.001$). Table 5 shows the estimates of mean complexity loss for each functional level (robust, prefrail, and frail) for the two scales.

**Table 5:** Descriptive statistics for the complexity of the fluctuations of hand movements across different frailty scores, namely, robust (left), prefrail (center), and frail (right)

	Robust	Prefrail	Frail
FRAIL scale	0.113 (0.002)	0.122 (0.002)	0.154 (0.008)
	CI = (0.110, 0.116)	CI = (0.117, 0.127)	CI = (0.137, 0.170)
Fried phenotype	0.111 (0.002)	0.127 (0.002)	0.153 (0.009)
	CI = (0.107, 0.115)	(0.124, 0.131)	(0.134, 0.171)

Results are reported for both the FRAIL scale (top) and the Fried phenotype (bottom) subsets. Average values, standard deviations, and confidence intervals for complexity loss are reported for each subset

Associations between frailty status and complexity loss for people over 65 years old were statistically significant for both the FRAIL scale (df = 2, Chisq = 39.743, p-value $< 0.001$) and the frailty phenotype (df = 2, Chisq = 27.545, p-value $< 0.001$). Table 6 shows the estimates of mean complexity loss for each frailty level (robust, prefrail, and frail) in adults 65 years old and older.

**Table 6:** Descriptive statistics for the complexity of the fluctuations of hand movements in older adults, across different frailty scores, namely, robust (left), prefrail (center), and frail (right)

	Robust	Prefrail	Frail
FRAIL scale	0.116 (0.003)	0.137 (0.004)	0.153 (0.010)
	CI = (0.110, 0.122)	CI = (0.130, 0.145)	CI = (0.131, 0.175)
Fried phenotype	0.115 (0.003)	0.134 (0.002)	0.153 (0.009)
	CI = (0.110, 0.122)	(0.129, 0.139)	(0.133, 0.172)

Results are reported for both the FRAIL scale (top) and the Fried phenotype (bottom) subsets. Average values, standard deviations, and confidence intervals for complexity loss are reported for each subset

Table 7 shows the results of post-hoc pairwise comparisons of complexity loss across frailty levels; p-values are reported after applying the Bonferroni correction. Complexity loss in the robust group resulted significantly different from complexity loss in both the prefrail and frail groups. However, no differences were observed between the complexity losses in the prefrail and frail groups.

**Table 7:** Results for the statistical differences between the complexity loss of older adults’ hand movements across different frailty scores, namely, robust vs. prefrail (left), robust vs. frail (center), and prefrail vs. frail (right)

	Robust vs. prefrail	Robust vs. frail	Prefrail vs. frail
FRAIL scale	t = 4.957, df = 31, p $<0.001$	t = 3.359, df = 31, p = 0.002	t = 1.436, df = 31, p = 0.483
Fried phenotype	t = 3.994, df = 31, p = 0.001	t = 3.817, df = 31, p = 0.002	t = 1.826, df = 31, p = 0.232

Results are reported for both the FRAIL scale (top) and the Fried phenotype (bottom) subsets

### Goodness of fit and predictive performance of the logistic models

Table 8 summarizes the final FRAIL model. The overall model is significant according to both a multivariable Wald test (W = 14.0, df = 2, p-value = 0.001) and an adjusted Wald test [F(2, 30) = 6.758, p-value = 0.004]. Only multimorbidity resulted statistically significant; however, we also retained complexity loss because it is the main variable in our study. Their interaction was not significant; thus, we retained the main effects model as the final FRAIL model. The probabilities estimated by this model did not fit well the probabilities observed in the data set [F(8, 23) = 5.000, p-value = 0.001]. Accordingly, the area under the curve showed poor discrimination power (AUC = 0.62).

**Table 8:** Logistic regression model for the FRAIL scale subset in the older sample

	Coefficient	Std Err	t	p-value
Multimorbidity	0.300	0.119	2.513	0.018*
DALPHA${}^{\text {a}}$	5.306	3.059	1.734	0.093
Intercept	0.053	0.391	0.136	0.893

The model includes multimorbidity and complexity loss without any nonlinear transformations and no interaction terms

*Statistically significant values (p $< 0.05$)

$${}^{\text {a}}$$Complexity loss

The final FRIED model is summarized in Table 9. The overall model is significant according to both a multivariable Wald test (W = 64.8, df = 4, p-value = 0.000) and an adjusted Wald test [F(4, 29) = 14.677, p-value = 0.000]. The coefficients of all four variables (sex, age, multimorbidity, and complexity loss) resulted statistically significant. The interactions of complexity loss with the other variables were not significant; thus, we retained the main effects model as the final FRIED model. The probabilities estimated by the FRIED model fitted well the probabilities observed in the data set [F(8, 24) = 0.466, p-value = 0.868] with the Archer–Lemeshow test showing high power (n = 1279; n1/n = 0.57). However, the discrimination power of the resulting classifier, although close to acceptable, remained poor (AUC = 0.69). In addition, a classifier based just on sex, age, and multimorbidity does not perform much worse (AUC = 0.67).

**Table 9:** Logistic regression model for the Fried phenotype subset in the older sample

	Coefficient	Std Err	t	p-value
Sex	0.538	0.133	4.050	0.000*
Age	0.059	0.015	4.042	0.000*
Multimorbidity	0.384	0.085	4.531	0.000*
DALPHA${}^{\text {a}}$	5.418	1.276	4.245	0.000*
Intercept	$-6.087$	1.200	$-5.071$	0.000*

The model includes all four variables (sex, age, multimorbidity, and complexity loss) without any nonlinear transformations and no interaction terms

*Statistically significant values (p $< 0.05$)

$${}^{\text {a}}$$Complexity loss

Finally, Table 10 shows the odds ratios for women over men, increments of five years in age, each additional comorbidity, and increments of 0.038 in complexity loss (which corresponds to the difference observed in the older population between the mean complexity-losses of the robust and the frail groups when accounting for three levels of frailty).

**Table 10:** Odds ratios for women over men, increments of 5 years in age, each additional comorbidity, and increments of 0.038 in complexity loss

	Increments	Odds ratio	95% CI
Sex	Women	1.712	(1.452, 1.972)
Age	5 Years	1.345	(1.201, 1.488)
Multimorbidity${}^{\text {a}}$	1 Disease	1.468	(1.302, 1.634)
DALPHA${}^{\text {b}}$	0.038	1.229	(1.134, 1.324)

$${}^{\text {a}}$$Multimorbidity

$${}^{\text {b}}$$Complexity loss

## Discussion

In this representative sample of the US population, we have shown that the movements of the non-dominant hand follow a fractal-like behavior, and that, in older people, their complexity loss is associated in a statistically significant way with being prefrail or frail according to the frailty phenotype; the diagnostic accuracy for frailty of a model with age, sex, multimorbidity and LoC was very close to be acceptable, but this latter variable contributed very little.

The distribution of frailty for older adults in the Fried phenotype subset is consistent with the prevalence figures reported by Fried et al. [7] in their original study. However, NHANES 2011–2014 did not test for gait speed and we had to use the participants’ responses to a questionnaire entry to assess the slowness component. The said questionnaire entry showed low sensitivity, especially for people younger than 65, thus, the results for that age group are less reliable. On another level, NHANES 2011–2014 holds a very large proportion of people over 65 scoring in the low-PA component. In particular, the number of people reporting no activity at all is way over the lower quintile threshold reported by Fried et al. [7]. Consequently, our estimations for frailty prevalence in NHANES 2011–2014 resulted slightly greater than Fried et al.’s [7]. Our estimations for the FRAIL scale subset resulted reasonable as well. The agreement between both scales is 67.8% and no one is two categories away from his frailty score in the other scale. Frailty and prefrailty are less prevalent according to the FRAIL scale, even for the subset of older people. This result is consistent with previous comparisons [48, 49]. However, the final logistic model based on the FRAIL scale subset does not show any significant associations with age, which casts doubts on the ability of the FRAIL scale to detect frail individuals in this sample.

The linearity of the log-log fluctuations of hand movements across different time scales is excellent (over 0.98) in both the FRAIL scale and the Fried phenotype subsets. These results suggest that hand movement fluctuations conform to a power law; which is supported by their narrow 95% CIs (0.07% and 0.09%, respectively). Therefore, hand movement fluctuations show a self-affine and scale-free nature with long-range correlations over multiple time scales. Moreover, goodness-of-fit did not show any dependencies with age, frailty level, or the value of the exponent. This means the fractal and complex nature of the process and its variability does not depend on these factors. Thus, the exponent of the corresponding power law can be used to characterize the fractal complexity of hand movements regardless of age or frailty level.

The same fractal-like behavior has been previously observed for the DFA outcomes of trunk acceleration signals recorded with wearable devices on older people’s waists [35] . Li et al. studied the complexity of the fluctuations of hand movements but they did not conduct any statistical tests to assess fractal behavior in their signals. They demonstrated self-similarity by providing a visual comparison of the irregularity of the fluctuation patterns of one of their signals at different temporal scales and comparing them with the irregularities of a shuffled version of the same signal [34].

Our logistic model for the Fried phenotype subset shows significant associations between frailty and sex, age, and multimorbidity. The observed associations imply that the probability of being frail is greater for women than for men and increases with age and with the number of comorbidities. These results are consistent with the previous established knowledge in the field and, thus, supports the use of the Fried phenotype subset for the study of frailty-related issues in the NHANES 2011–2014 data set. The Fried phenotype model also shows a significant association between frailty and complexity loss after adjusting for sex, age, and multimorbidity. The observed association imply that the probability of being frail increases for larger reductions in complexity. This result is consistent with the LoC model, which states that either a noisier or a more regular behavior in physiological processes result in a decrease of system adaptability. Li et al., conducted a longitudinal follow-up study and, in the same line, they observed a statistically significant increase in the risk of becoming frail or developing a disability for individuals with less complex fluctuations of hand movements at baseline [34]. They also observed that the increased risk of frailty remained statistically significant after adjusting for age, sex, education, and multimorbidity [34]. We observed a lack of significance in an unadjusted, pairwise comparison between the prefrail and the frail groups, but a statistically significant difference between the healthy (aka robust) and non-healthy (aka prefrail and frail) groups. This result suggests that complexity loss could help to identify those in need of a therapeutical intervention but not to assess the severity of functional decline. However, the performance of the model as a binary classifier (robust vs. prefrail/frail) does not reach an acceptable level, although it gets very close (AUC = 0.69; threshold for acceptable performance = 0.70). Moreover, the small improvement in the AUC value due to complexity loss suggests that its inclusion in the model is not worth the increment in cost and effort respect to a model based on sex, age, and multimorbidity.

Other studies on the complexity of accelerometry signals from wearable devices present some methodological differences compared to the present study. Most of them assess the complexity of trunk movements instead of hand movements. Rector et al. collected signals with an accelerometer on the participants’ chests [23]; Paraschiv-Ionescu et al. and Zhang et al. used an accelerometer on the participants’ lower backs [33, 36]; and Raichlen et al. used an accelerometer on the participants’ waists [35]. The only study we found on hand movements was Li et al.’s [34]. Paraschiv-Ionescu et al. and Zhang et al. did not assessed the complexity of time series of activity counts but the complexity of a multivariate metric based on the type, duration, and intensity of PA [33, 36]. Rector et al., Raichlen et al., and Li et al. each used epochs of different duration to compute their time series of activity counts; none of them used MIMS. Rector et al. used 4 s epochs [23]; Raichlen et al. used 1 min epochs [35]; and Li et al. used 15 s epochs [34]. Different studies have also used different complexity measurements. Paraschiv-Ionescu et al. and Zhang et al. used Lempel-Ziv complexity [33, 36]; Rector et al. used MSE [23]; while Raichlen et al. and Li et al. used DFA like we did in the present study [34, 35]. Finally, different studies have used different scales to operationalize the functional status of older adults. Zhang et al. used the Community Balance and Mobility Scale (CBMS) [36]; Rector et al. used The Older Persons and Informal Caregivers Survey—Minimal Dataset (TOPICS-MDS) [23]; while the scale in Li et al.’s work was based on the Linda Fried phenotype like we did in the present study [34]. Despite these methodological differences, all the studies found an association between the LoC in accelerometry signals from wearable devices and functional decline.

The main limitation of the present study came from the fact that the original NHANES 2011–2014 wave did not include the clinical assessment of frailty. For some of the criteria in the FRAIL scale and the Fried phenotype, the data set did not include the original variables needed to score the items. Nevertheless, the variables in the data set allowed us to compute indirect estimations for those criteria. As discussed above, despite the noise added by these indirect estimations, the distribution of the resulting outcomes was consistent with the previous established knowledge in the field. Another limitation came from the cross-sectional nature of the data set; which limited the scope of our study to between-subjects differences and did not allow us to explore the ability of complexity loss to detect changes in function over time. However, the study conducted by Zhang et al. suggests that such a relationship exists since they observed an association between the changes in the complexity of their multivariate metric and functional changes measured with the CBMS [36]. On the other hand, the large size of the sample strengthens the power of our results. Finally, the main strength of the present study is the availability of accelerometry and angular velocity measures of the hand movements for a nationally-representative sample.

## Conclusion

Frailty can be characterized in the NHANES 2011–2014 data set with the Fried phenotype. The movement of a person’s non-dominant hand in free-living conditions is a fractal process regardless of age or frailty level and its self-similarity can be quantified with the exponent of a power law. Higher levels of complexity loss are associated with higher levels of frailty. However, the effect of this association is not strong enough to justify the use of complexity loss to detect frailty in older adults after adjusting for sex, age, and multimorbidity.

## Figures and Tables

**Fig. 4:**
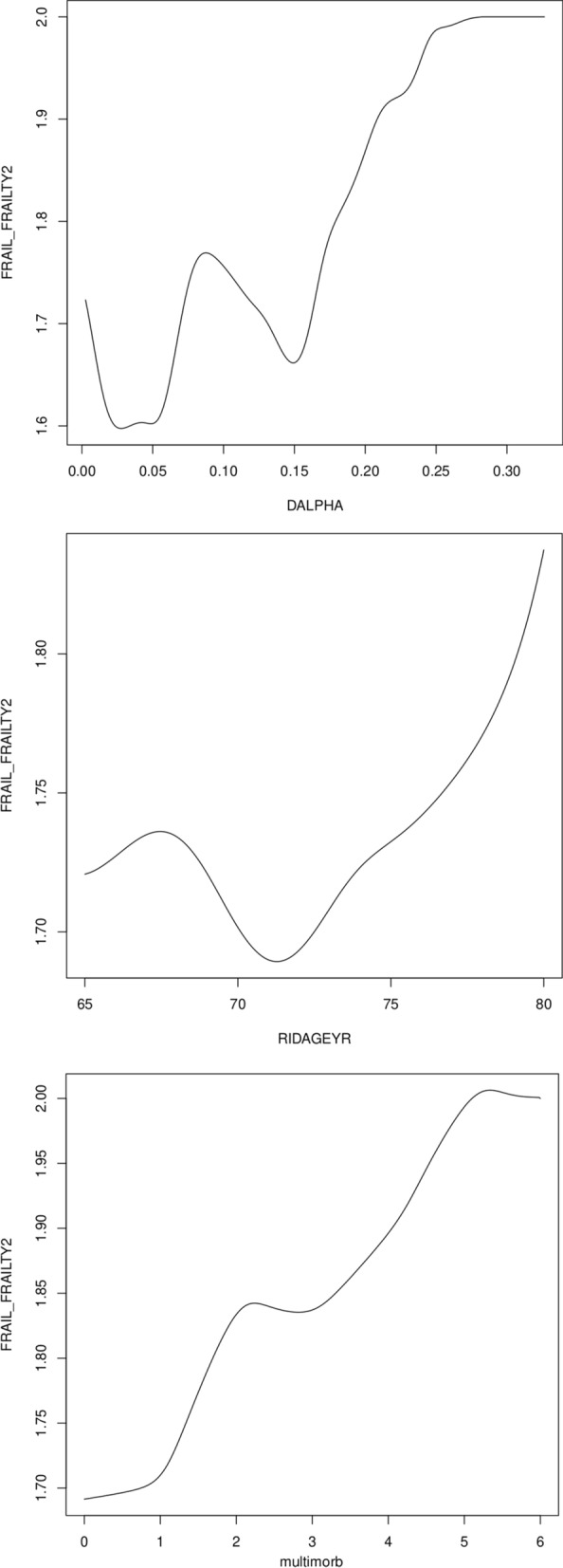
Nonlinear behavior of frailty respect to the continuous variables in the FRAIL model. Frailty vs. complexity loss (top), frailty vs. age (center), and frailty vs. Multimorbidity (bottom)

**Fig. 5:**
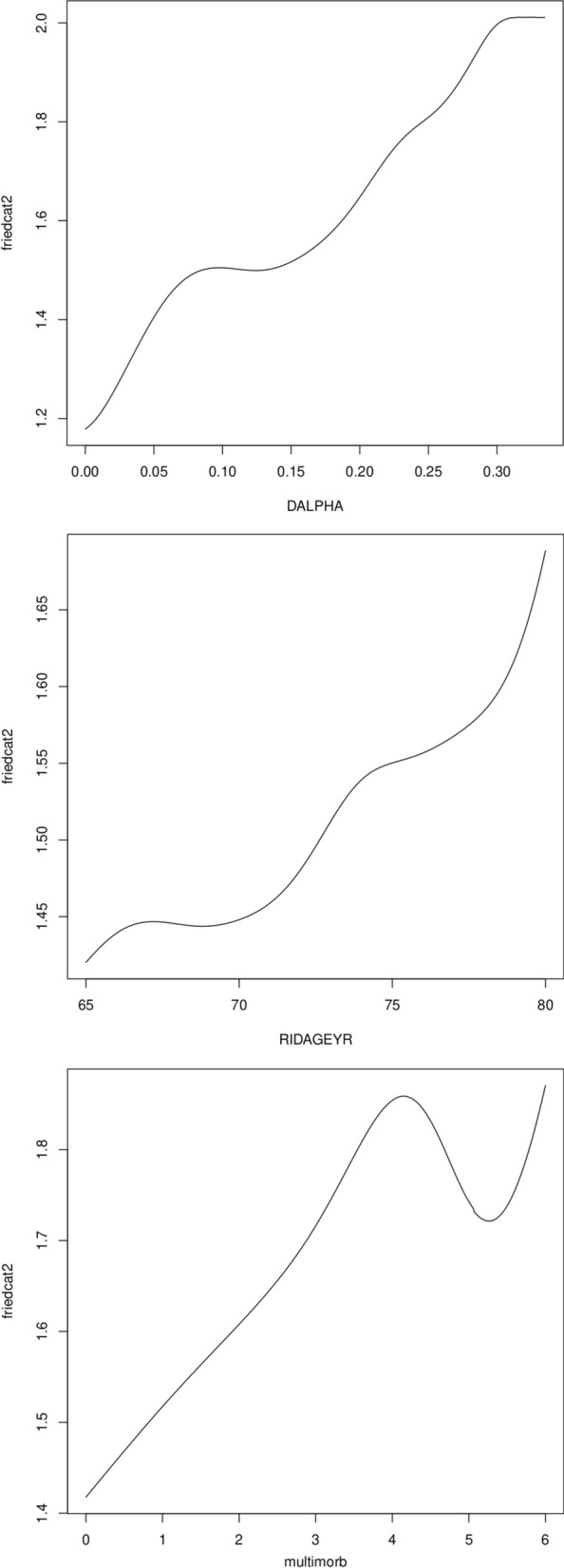
Nonlinear behavior of frailty respect to the continuous variables in the FRIED model. Frailty vs. complexity loss (top), frailty vs. age (center), and frailty vs. multimorbidity (bottom)

## Appendix: Building the models

### The FRAIL model

The smoothed scatter plots for the FRAIL model in Fig. 4 show nonlinear behaviors of frailty status with both complexity loss and age.

Both complexity loss and age were initially modeled by fitting quadratic and linear terms for each of them after centering the variables. Both the quadratic and linear terms resulted non-significant for age. In contrast, a linear model for complexity loss (DALPHA) was statistically significant. The addition of the quadratic term (DALPHA_square) resulted in non-significant coefficients. According to the smoothed scatterplot, only a linear model was fit for multimorbidity. The corresponding coefficient resulted statistically significant. The results of all these models are summarized in Table 11.

**Table 11:** Results of the univariate regressions for the continuous variables in the FRAIL model

	Coefficient	Std Err	t	p-value
DALPHA${}^{\text {a}}$	6.574	3.655	1.799	0.082
DALPHA_square${}^{\text {b}}$	65.723	38.941	1.688	0.102
Intercept	0.882	0.184	4.70	0.000*
DALPHA${}^{\text {a}}$	6.403	3.064	2.090	0.045*
Intercept	0.222	0.412	0.539	0.594
Age	0.029	0.029	0.999	0.326
Age_square${}^{\text {c}}$	0.009	0.007	1.261	0.218
Intercept	0.843	0.216	3.904	0.001*
Age	0.034	0.027	1.255	0.219
Intercept	$-1.429$	1.975	$-0.723$	0.475
Multimorbidity	0.345	0.115	2.985	0.000*
Intercept	0.695	0.152	4.568	0.000*

*Statistically significant values (p $< 0.05$)

$${}^{\text {a}}$$Complexity loss

${}^{\text {b}}$Quadratic term for complexity loss

${}^{\text {c}}$Quadratic term for age

Table 12 shows the results for a FRAIL model comprising sex, age, and multimorbidity as covariates. Only multimorbidity showed a significant coefficient. The addition of a quadratic term for age did not result in any significant changes in the model.

**Table 12:** Results of an intermediate FRAIL model comprising the effects sex, age, and multimorbidity

	Coefficient	Std Err	t	p-value
Sex	0.454	0.285	1.592	0.123
Age	0.029	0.026	1.117	0.274
Multimorbidity	0.331	0.116	2.846	0.008*
Intercept	$-2.116$	1.800	$-1.175$	0.250
Sex	0.440	0.290	1.516	0.141
Age	0.024	0.028	0.877	0.388
Age _square${}^{\text {a}}$	0.009	0.007	1.188	0.245
Multimorbidity	0.331	0.115	2.888	0.008*
Intercept	$-0.165$	0.462	$-0.358$	0.723

*Statistically significant values (p $< 0.05$)

${}^{\text {a}}$Quadratic term from age

Table 13 shows the results for a FRAIL model comprising sex, age, multimorbidity, and complexity loss as covariates. Multimorbidity remains the only significant term in the model.

**Table 13:** Results of an intermediate FRAIL model comprising the effects sex, age, multimorbidity, and complexity loss

	Coefficient	Std Err	t	p-value
Sex	0.464	0.295	1.575	0.127
Age	0.024	0.028	0.855	0.400
Multimorbidity	0.292	0.121	2.418	0.023*
DALPHA${}^{\text {a}}$	5.158	3.120	1.654	0.110
Intercept	$-2.387$	1.842	$-1.296$	0.206
Sex	0.483	0.291	1.657	0.110
Age	0.028	0.029	0.975	0.338
Multimorbidity	0.314	0.129	2.435	0.022*
DALPHA${}^{\text {a}}$	4.966	3.887	1.278	0.213
DALPHA_square${}^{\text {b}}$	74.239	38.016	1.953	0.062
Intercept	$-2.220$	1.961	$-1.132$	0.268

*Statistically significant values (p $< 0.05$)

$${}^{\text {a}}$$Complexity loss

${}^{\text {b}}$Quadratic term for complexity loss

Finally, Table 14 shows the results for a FRAIL model excluding the variables age and sex that were not significant. The coefficients for complexity loss and multimorbidity varied by less than a 10%; multimorbidity maintained its statistical significance and complexity loss remained non-significant. Adding a quadratic term for complexity loss did not result in a significant coefficient.

**Table 14:** Results of two FRAIL models comprising the effects multimorbidity and complexity loss

	Coefficient	Std Err	t	p-value
Multimorbidity	0.300	0.119	2.513	0.018*
DALPHA${}^{\text {a}}$	5.306	3.059	1.734	0.093
Intercept	0.053	0.391	0.136	0.893
Multimorbidity	0.316	0.125	2.529	0.017*
DALPHA${}^{\text {a}}$	5.299	3.736	1.418	0.167
DALPHA_square${}^{\text {b}}$	70.363	39.425	1.785	0.085
Intercept	0.542	0.211	2.562	0.016*

*Statistically significant values (p $< 0.05$)

$${}^{\text {a}}$$Complexity loss

${}^{\text {b}}$Quadratic term for complexity loss

The interaction between multimorbidity and complexity loss resulted non-significant (coefficient = 4.629, std err = 2.449, t = 1.890, p-value = 0.069).

### The FRIED model

The smoothed scatter plots for the FRIED model in Fig. 5 show nearly linear behavior for complexity loss. However, the age and multimorbidity smoothed scatter plot suggest that a nonlinear transformation might be appropriate.

Both complexity loss and multimorbidity were initially modelled with a linear term. A univariable linear model for multimorbidity was fit and was statistically significant. Three additional models were fit with different nonlinear transformations of multimorbidity, one for a logarithmic transformation, a second one for a transformation of the form $1 - 1/multimorbidity$ (both statistically significant), and another one comprising a spline with a knot at 4, where only the coefficient below 4 was significant. The AIC resulted lower in the linear model (1715.657 vs. 1720.248 vs. 1727.860 vs. 1717.615, respectively). The value of t was more extreme for the linear model as well (see Table 15). The linear model for multimorbidity was retained. Age was initially modeled with a linear and quadratic model. The linear term resulted statistically significant but the quadratic term did not, although the quadratic model resulted in a lower value of the AIC (1734.774 vs. 1736.533). The linear model for age was retained. The results of these four models are summarized in Table 15.

**Table 15:** Results of the univariate regressions for the continuous variables in the FRIED model

	Coefficient	Std Err	t	p-value
DALPHA${}^{\text {a}}$	6.810	1.342	5.075	0.000*
Intercept	$-0.790$	0.203	$-3.900$	0.000*
Age	0.066	0.015	4.291	0.000*
Age_square${}^{\text {b}}$	0.005	0.003	1.835	0.076
Intercept	$-0.049$	0.121	$-0.407$	0.687
Multimorbidity	0.415	0.086	4.825	0.000*
Intercept	$-0.341$	0.133	$-2.558$	0.016*
log(Multimorb${}^{\text {c}}$)	0.824	0.177	4.648	0.000*
Intercept	$-0.391$	0.145	$-2.702$	0.011*
$1 - 1$/Multimorb${}^{\text {c}}$	1.299	0.300	4.331	0.000*
Intercept	$-0.392$	0.150	$-2.620$	0.014*
Morb.ns1${}^{\text {d}}$	3.297	0.867	3.804	0.001*
Morb.ns2${}^{\text {e}}$	1.959	1.277	1.534	0.136
Intercept	$-0.335$	0.137	$-2.448$	0.020*

*Statistically significant values (p $< 0.05$)

$${}^{\text {a}}$$Complexity loss

${}^{\text {b}}$Quadratic term for age

$${}^{\text {c}}$$Multimorbidity

${}^{\text {d}}$Coefficient of the spline below the knot at 4

${}^{\text {e}}$Coefficient of the spline above the knot at 4

Table 16 shows the results for an intermediate FRIED model comprising sex and age as covariates. The coefficients for both sex and age were statistically significant. Adding a quadratic term for age kept sex and age significant; but the quadratic term was not significant.

**Table 16:** Results of an intermediate FRIED model comprising the effects of sex and age

	Coefficient	Std Err	t	p-value
Sex	0.445	1.328	3.346	0.002*
Age	0.068	0.016	4.336	0.000*
Intercept	$-5.505$	1.248	$-4.413$	0.000*
Sex	0.437	0.136	3.208	0.003*
Age	0.066	0.016	4.225	0.000*
Age_square${}^{\text {a}}$	0.005	0.003	1.637	0.112
Intercept	$-0.696$	0.257	$-2.712$	0.011*

*Statistically significant values (p $< 0.05$)

${}^{\text {a}}$Quadratic term for age

Table 17 shows the results for another intermediate FRIED model comprising sex and age and adding multimorbidity to the set of covariates. The coefficients for all three covariates (sex, age, and multimorbidity) were statistically significant and the addition of multimorbidity showed a confounding effect on sex. Adding a quadratic term for age did not result statistically significant.

**Table 17:** Results of an intermediate FRIED model comprising the effects of sex, age, and multimorbidity

	Coefficient	Std Err	t	p-value
Sex	0.540	0.136	3.975	0.000*
Age	0.062	0.015	4.081	0.000*
Multimorbidity	0.422	0.084	5.049	0.000*
Intercept	$-5.676$	1.208	$-4.697$	0.000*
Sex	0.533	0.139	3.827	0.001*
Age	0.060	0.016	3.892	0.001*
Age _square${}^{\text {a}}$	0.006	0.003	1.774	0.087
Multimorbidity	0.428	0.085	5.058	0.000*
Intercept	$-1.292$	0.302	$-4.273$	0.000*

*Statistically significant values (p $< 0.05$)

${}^{\text {a}}$Quadratic term for age

Table 18 shows the results for a complete FRIED model comprising sex, age, multimorbidity, and complexity loss as covariates. The coefficients for all four covariates (sex, age, multimorbidity, and complexity loss) were statistically significant. Adding a quadratic term for age did not result statistically significant.

**Table 18:** Results of a complete FRIED model comprising the effects of sex, age, multimorbidity, and complexity loss (DALPHA)

	Coefficient	Std Err	t	p-value
Sex	0.538	0.133	4.050	0.000*
Age	0.059	0.015	4.042	0.000*
Multimorbidity	0.384	0.085	4.531	0.000*
DALPHA${}^{\text {a}}$	5.418	1.276	4.245	0.000*
Intercept	$-6.087$	1.200	$-5.071$	0.000*
Sex	0.531	0.136	3.902	0.001*
Age	0.057	0.015	3.831	0.001*
Age_square${}^{\text {b}}$	0.006	0.003	1.789	0.085
Multimorbidity	0.390	0.086	4.548	0.000*
DALPHA${}^{\text {a}}$	5.453	1.279	4.263	0.000*
Intercept	$-1.945$	0.383	$-5.078$	0.000*

*Statistically significant values (p $< 0.05$)

$${}^{\text {a}}$$Complexity loss

${}^{\text {b}}$Quadratic term for age

Table 19 shows the results for the interaction terms in the FRIED model. None of the interaction terms is statistically significant.

**Table 19:** Results of a complete FRIED model comprising the effects of sex, age, multimorbidity, complexity loss and their interaction terms

	Coefficient	Std Err	t	p-value
Sex	0.542	0.132	4.090	0.000*
Age	0.058	0.015	3.959	0.001*
Multimorbidity	0.389	0.086	4.519	0.000*
DALPHA${}^{\text {a}}$	11.236	3.684	3.050	0.005*
Sex $\times$ DALPHA${}^{\text {a}}$	$-3.945$	2.418	$-1.632$	0.115
Age $\times$ DALPHA${}^{\text {a}}$	$-0.036$	0.277	$-0.131$	0.897
morb${}^{\text {b}}$ $\times$ DALPHA${}^{\text {a}}$	$-0.633$	1.224	$-0.517$	0.610
Intercept	$-0.735$	0.234	$-3.147$	0.004*

*Statistically significant values (p $< 0.05$)

$${}^{\text {a}}$$Complexity loss

$${}^{\text {b}}$$Multimorbidity
